# Biomimetic In Vitro Model of Cell Infiltration into Skin Scaffolds for Pre-Screening and Testing of Biomaterial-Based Therapies

**DOI:** 10.3390/cells8080917

**Published:** 2019-08-17

**Authors:** Rafael Ballesteros-Cillero, Evan Davison-Kotler, Nupur Kohli, William S. Marshall, Elena García-Gareta

**Affiliations:** 1 Regenerative Biomaterials Group, RAFT Institute, Mount Vernon Hospital, Northwood HA6 2RN, UK; 2 Biology Department, St. Francis Xavier University, Antigonish, NS B2G 2W5, Canada

**Keywords:** cell infiltration, biomimetic, wound healing, tissue engineering, skin substitute, biomaterial

## Abstract

Due to great clinical need, research where different biomaterials are tested as 3D scaffolds for skin tissue engineering has increased. In vitro studies use a cell suspension that is simply pipetted onto the material and cultured until the cells migrate and proliferate within the 3D scaffold, which does not mimic the in vivo reality. Our aim was to engineer a novel biomimetic in vitro model that mimics the natural cell infiltration process occurring in wound healing, thus offering a realistic approach when pre-screening and testing new skin substitutes. Our model consists of porous membrane cell culture inserts coated with gelatin and seeded with human dermal fibroblasts, inside which two different commercially available dermal substitutes were placed. Several features relevant to the wound healing process (matrix contraction, cell infiltration and proliferation, integration of the biomaterial with the surrounding tissue, and secretion of exogenous cytokines and growth factors) were evaluated. Our results showed that cells spontaneously infiltrate the materials and that our engineered model is able to induce and detect subtle differences between different biomaterials. The model allows for room for improvements or “adds-on” and miniaturization and can contribute to the development of functional and efficient skin substitutes for burns and chronic wounds.

## 1. Introduction

Over the last two decades, there has been a significant increase in the number of studies in which different biomaterials are tested as 3D scaffolds in tissue engineering, and skin substitutes have not been an exception [1]. The current range of skin substitutes is as numerous as the possible combinations of their characteristics, since these are not mutually exclusive. These characteristics include, but are not limited to, cellularity (acellular or cellular), layering (single layer or bilayer), replaced region (epidermis, dermis, or both), materials used (natural, synthetic, or both), and permanence (temporary or permanent) [1,2]. Many skin substitutes are already available in the market and although all of them have their advantages and disadvantages, to this date, a complete functional skin substitute has not been produced [3].

**Figure 1:**
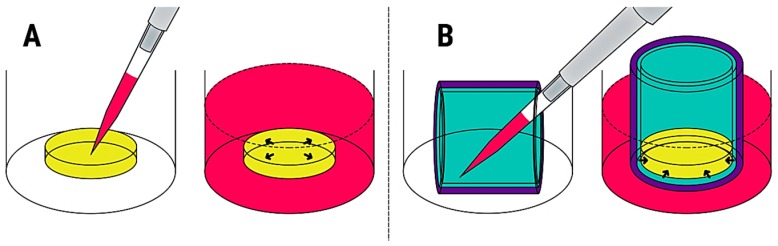
Comparison between static seeding and our proposed model. (**A**) Static seeding: a cell suspension is pipetted on top of the biomaterial, cells are incubated and allowed to attach to the scaffold. Fresh medium is then added. Finally, the cells migrate and proliferate within the scaffold. (**B**) In vitro model of wound healing: a porous membrane cell culture insert is coated with gelatin onto which human dermal fibroblasts (HDF) are later seeded. After allowing attachment, cells are covered with fresh medium and cultured for 1 h. The scaffold is placed at the bottom of the insert and fresh medium added. Finally, the HDF advance from the walls and the bottom of the insert to the biomaterial, thus mimicking the natural cell infiltration process of wound healing. Colour key: pink, cell culture medium (contains cells when in the pipette); purple, porous membrane cell culture insert; turquoise, gelatin coating and HDF; yellow, biomaterial. Arrows indicate the direction of cell infiltration and spreading.

Developing new skin scaffolds requires in vitro testing before in vivo experimentation in animal models and ultimately clinical trials in humans [4]. In the specific case of in vitro wound healing studies, cells are typically cultured on a biomaterial in static conditions, where a cell solution is simply pipetted onto the biomaterial and cultured until the cells migrate and proliferate within the 3D scaffold (Figure 1A). However, the current form of static in vitro study is far from the in vivo reality and can lead to incorrect assumptions pertaining to the in vivo outcomes of scaffold implantation, such as integration, cellularization, and contraction [4].

**Figure 2:**
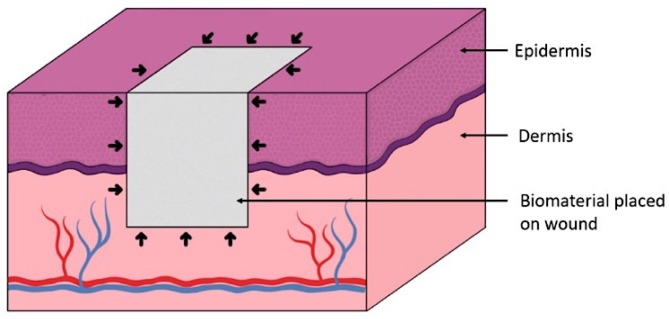
Scheme of the in vivo process of cell infiltration (black arrows) from the wound bed and edges into a skin scaffold.

In vivo, there is a dynamic process of cell migration into the scaffold from the wound edges and bed (Figure 2). The aim of this study was to engineer a novel biomimetic in vitro model for the pre-screening and testing of new skin substitutes under development. Our proposed in vitro model mimics the natural cell infiltration process into 3D scaffolds occurring during wound healing, thus offering a realistic approach when testing novel skin biomaterials (Figure 1B).

Our engineered model consists of porous membrane cell culture inserts coated with gelatin and seeded with human dermal fibroblasts (HDF) (Figure 1B). This represents the healthy tissue surrounding a wound. Gelatin is a partially hydrolyzed version of collagen where the triple-helical structure of collagen is changed into single-stranded molecules. Gelatin is advantageous over collagen as it is more economical and allows enhanced cell adhesion because of the abundant presence of arginine-glycine-aspartic acid (RGD) sequences [5]. Dermal fibroblasts are the main cell type found in dermal tissue [6]. In this model, the cells can advance from the wall and bottom of the insert instead of being pipetted on top of the scaffold, thus mimicking the natural cell infiltration process that happens during wound healing. Results achieved with this model would be more representative of the in vivo scenario and assist in the development of more effective biomaterial-based therapies for skin repair.

## 2. Materials and Methods

### 2.1. Inserts and Gelatin Coating

Polycarbonate cell culture-treated inserts (140629, Thermo Scientific, Denmark) with 8 µm pore size in 24-well plate were used. Bottom of inserts consisted of a polycarbonate porous membrane with 0.47 cm^2^ culture area.

A 0.1% *w*/*v* solution from bovine skin gelatin (Type B, G9391, Sigma-Aldrich, Gillingham, UK) was prepared in distilled water, warmed up for 1 h at 37 °C and 0.2 µm sterile-filtered. Inserts were then placed in a 24-multiwell plate where they were covered with the gelatin solution. The plate with the inserts was stored at 4 °C in the dark until used.

### 2.2. Coomassie Blue Staining of Inserts

A Coomassie blue staining of the inserts was carried out to confirm visually presence of gelatin coating [7,8]. Inserts (*n* = 3) were stained using Bradford Ultra™ reagent (BFU05L, Expedeon, Cambridge, UK), a Coomassie-binding, colorimetric reagent that shifts colour from brown to blue when bound to protein. Inserts were placed in 5 mL sterile tubes, 3 mL of Bradford Ultra™ reagent were added per tube, which were left to stand at room temperature (set at 20 °C) for 1 h. The Bradford Ultra™ reagent was removed and inserts washed once with distilled water to eliminate excess dye.

### 2.3. Cell Culture

Primary normal human dermal fibroblasts (ATCC, Lot# 80616174) were used in our study. Cells were cultured in Dulbecco’s modified Eagle’s medium (DMEM, 31885-023, Gibco, Paisley, UK) supplemented with 10% fetal bovine serum (FBS, 10270-106, Gibco, Paisley, UK), 100 U/mL penicillin and streptomycin (15140-122, Gibco, Paisley, UK), and 100 µM L-glutamine (25030-024, Gibco, Paisley, UK) at 37 °C, 5% CO_2_. Medium was changed every 3 days. Cells were used at passage 8, when they were still proliferative. Morphology of HDF cultures was regularly observed under a phase-contrast light microscope (Nikon Eclipse TS100, Nikon, Tokyo, Japan).

### 2.4. Cell Seeding

Before cell seeding, excess gelatin was removed from the inserts, which were then washed with PBS and placed horizontally in a 12-multiwell plate. A cell suspension of 2 × 10^6^ cells/mL was prepared. 20 µL of the cell suspension were pipetted on one of the inserts’ sides and allowed incubation for 10 min at 37 °C with 5% CO_2_. After incubation, the inserts were turned 180° and 20 µL of the cell suspension pipetted on the opposite side to the original seeding point. Inserts were incubated for 10 min at 37 °C with 5% CO_2_ and the same steps already described were repeated on the other two sides of the insert, but with 8 min of incubation after each seeding step. The inserts were then placed in their upward position, 20 µL of cell suspension added to the inserts’ bottom and incubated for 8 min at 37 °C with 5% CO_2_. Therefore, a total of 2 × 10^5^ cells were seeded per insert. Finally, 1 mL of warm medium was added per well and plates incubated for 1 h at 37 °C with 5% CO_2_ before application of the dermal biomaterials.

### 2.5. Skin Substitutes Used and Scanning Electron Microscopy (SEM)

MatriDerm^®^ (MedSkin Solutions Dr Suwelack AG, Billerbeck, Germany) is a commercially available dermal replacement scaffold comprising a lyophilized non-crosslinked porous layer (1 mm thickness) of bovine collagen type I, III and V coated with 3% bovine elastin hydrolysate [1,9].

PELNAC™ (Gunze Ltd., Tokyo, Japan) is a commercially available full-thickness skin replacement scaffold. PELNAC™ is a freeze-dried crosslinked sponge layer (3 mm thickness) of porcine tendon-derived atelocollagen type I (dermal component) with a perforated silicone membrane (epidermal component) [10,11,12]. Atelocollagen is a low-immunogenic derivative of collagen obtained by removal of N- and C-terminal telopeptide components with type I pepsin [13].

Specimens of both scaffolds were cut (5 mm × 5 mm), placed on stubs and sputter carbon-coated before observation (FEI Inspect F, Oxford Instruments, Oxford, UK).

### 2.6. Placement of the Biomaterial

1 mL pipette tips were cut at the tip to get 16 mm length pieces to act as “weights” to avoid flotation of the biomaterials. Weights were autoclaved prior to application. Pieces of the biomaterials were cut (11 mm diameter), sterilized with 70% IMS for 5 min and washed 3 times with PBS for 5 min each. One hour after ending cell seeding, the biomaterials were placed inside the inserts ensuring they were fully extended. PELNAC™ was placed with the sponge layer facing the bottom of the insert. The weights were placed on top of them and medium added. The medium was changed periodically.

### 2.7. Alamar Blue Assay

Alamar Blue [14] was used to monitor cell proliferation in the biomaterials as well as on the inserts. Alamar Blue was diluted 10 times in phenol-free medium. On days 3, 7 and 10, the medium from the multiwell plate was harvested and the biomaterials were transferred to empty wells. The inserts and the biomaterials (*n* = 4 per biomaterial/insert and time point) were washed once with PBS, 1 mL of the diluted Alamar Blue was added to the samples and empty wells as blank (*n* = 4) and incubated for 1 h at 37 °C with 5% CO_2_. Afterwards, 100 µL × 3 samples from each well were moved to a 96 well microplate (flat bottom) and their fluorescence was measured using a microplate reader (excitation filter at 544 nm, emission filter at 590 nm, FLUOstar Omega, BMG Labtech, Aylesbury, UK). The measurements were compared against a calibration curve (0 to 1 × 10^6^ cells, y = 0.0016x with R^2^ = 0.9835) to convert fluorescent intensity to cell number.

### 2.8. Matrix Contraction

Matrix contraction of the skin substitutes used was measured on days 3, 7 and 10 after cell seeding by taking pictures of the biomaterials (*n* = 4 per biomaterial and time point), accompanied by a ruler, using a digital camera. Photos were taken after the Alamar Blue assay. Results were presented as percentage of relative area.

### 2.9. Cell Infiltration into Skin Substitutes

**Figure 3:**
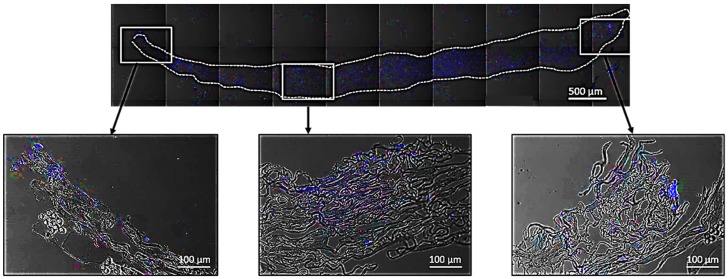
Top image shows an example of tiled-scanned confocal microphotograph of a 4 µm thick MatriDerm^®^ section stained with DAPI (blue fluorescence indicative of cell nuclei) used to assess cell infiltration into the scaffolds. The white dashed line indicates the edge of the scaffold. The three micrographs at the bottom show higher magnification images of cells (blue fluorescence) in the scaffold that is visible as a mesh of white/grey fibers.

Fixed specimens in 10% formalin (*n* = 3 per biomaterial and time point) were processed and cut into 4 µm sections that were deparaffinized, rehydrated and washed in distilled water before applying Fluoroshield™ with DAPI mounting media (F6057, Sigma, Gillingham, UK) as per the manufacturer’s instructions. The mounting media was left to set for 5 min at room temperature, the sections were then cover slipped and the edges sealed with nail varnish. Sections were imaged with a confocal scanning laser microscope (Leica DMIRE2, Leica, Wetzlar, Germany). Sections were tile-scanned for the entire XY plane of the scaffold and then merged using 5% overlap automated function of the confocal microscope (Figure 3).

3 different score systems were used to evaluate cell infiltration into the skin substitutes [8]: (1) Score 1, which evaluates distance migrated by the cells: 0, cells not adhered to scaffold; 1, cells adherent to scaffold surface but no penetrance; 2, cells have penetrated but do not go past the middle of the scaffold; 3, cells have penetrated past the middle of the scaffold but do not reach the other end; 4, total penetrance of cells through the scaffold depth. (2) Score 2, which evaluates cell density: 0, no cells adhered; 1, majority of cells on 1/4 depth of the scaffold; 2, majority of cells on half of the scaffold; 3, majority of cells on 3/4 depth of the scaffold; 4, cells evenly distributed throughout the scaffold depth. (3) Score 3, which evaluates both distance migrated and cell density: Score 1 × Score 2.

### 2.10. Secretion of Exogenous Cytokines and Growth Factors

On days 3, 7 and 10 of culture (*n* = 4 per biomaterial and time point) secretion of tumor necrosis factor alpha (TNFα), insulin-like growth factor 1 (IFG1), vascular endothelial growth factor (VEGF), interleukin 6 (IL-6), fibroblast growth factor b (FGFb), transforming growth factor beta (TGFβ), epidermal growth factor (EGF) and leptin was profiled (EA-1011, Signosis Inc, Sunnyvale, CA, USA). At days 3, 7 and 10 after seeding, medium was collected from the center of the insert and frozen at −80 °C. After thawing, supernatants (100 µL/well) were pipetted into coated wells and incubated for 1 h at room temperature with gentle shaking. Wells were washed 3 times with 200 µL wash buffer, 100 µL diluted biotin-labelled antibody mixture was added per well and incubated as before. After repeating the washing step, 100 µL of diluted streptavidin-HRP conjugate was added per well and incubated for 45 min at room temperature with gentle shaking. After washing, 100 µL of substrate was added per well, followed after 30 min incubation by stop solution (50 µL/well). Absorbance at 450 nm was measured using a microplate reader (FLUOstar Omega, BMG Labtech, Aylesbury, UK).

### 2.11. Statistical Analysis

Comparisons between groups were made using SigmaStat 3.5 software: one-way ANOVA with a Holm-Sidak post hoc analysis was carried out and a *p* < 0.05 was considered a significant result.

## 3. Results

### 3.1. Gelatin Coating and Cell Seeding

**Figure 4:**
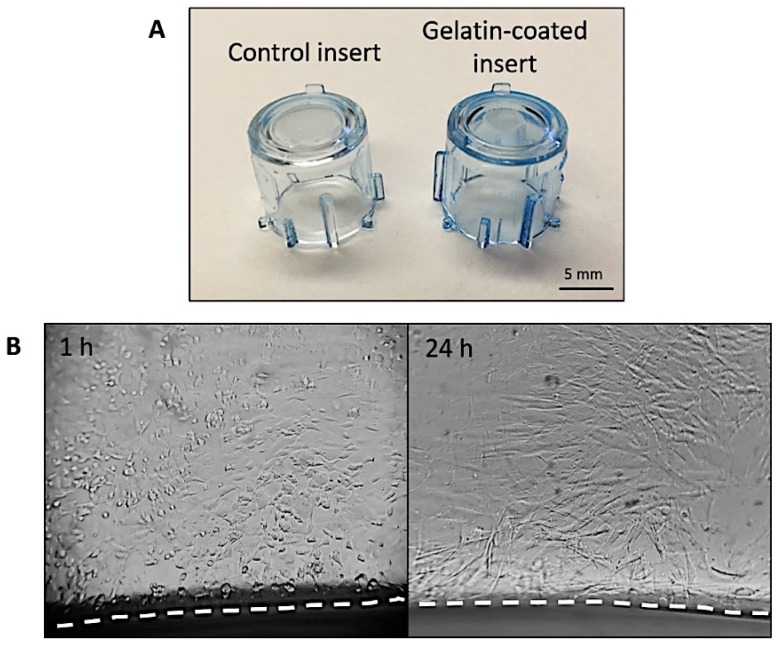
(**A**) Coomassie blue staining of uncoated (control) versus gelatin-coated inserts showing an increment in blue colour intensity for the latter. (**B**) Representative images of HDF on the walls of the insert 1 h and 24 h after seeding. The dashed line indicates the bottom of the insert. Images taken at 20X magnification. Note: as a result of the curvature and inclination of the insert it is only possible to correctly focus part of the field.

Coomassie blue staining showed that the inserts were evenly coated with gelatin on both the bottom and walls, as evidenced by the increase in blue color intensity between uncoated and gelatin-coated implants (Figure 4A). This was important to create an extracellular matrix (ECM)-like surface before cell seeding.

Optimisation of the rotating cell seeding technique allowed us to evenly seed cells on the walls and bottom of the gelatin-coated inserts. After 1 h of seeding, cells covered all surfaces and appeared rounded indicating that they were in the process of attaching to the gelatin coating. After 24 h of incubation, cells formed a monolayer that covered the walls and bottom of the inserts (Figure 4B).

### 3.2. SEM of Skin Substitutes

**Figure 5:**
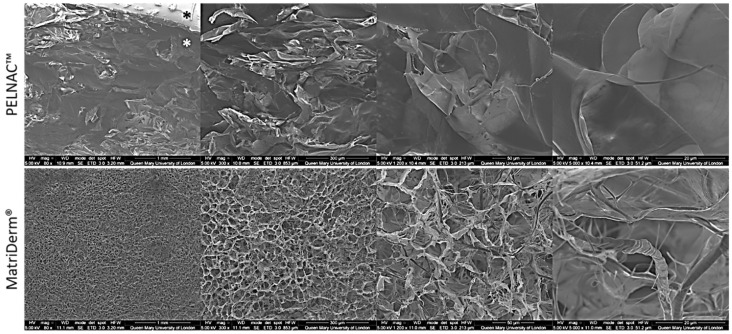
SEM images of commercially available skin scaffolds MatriDerm^®^ and PELNAC™ used in this study. Black * indicates the silicone membrane while white * indicates the sponge of PELNAC™.

SEM images showed that MatriDerm^®^ is a porous matrix with gradient porosity ranging from macro to micro interconnected pores. Bundles of fibres are seen at higher magnifications. In the case of PELNAC™, SEM showed that the sponge’s structure is different to the matrix of MatriDerm^®^. PELNAC™ also displays pores of different sizes that are interconnected (Figure 5).

### 3.3. Matrix Contraction and Integration of the Biomaterial with the Surrounding Tissue

**Figure 6:**
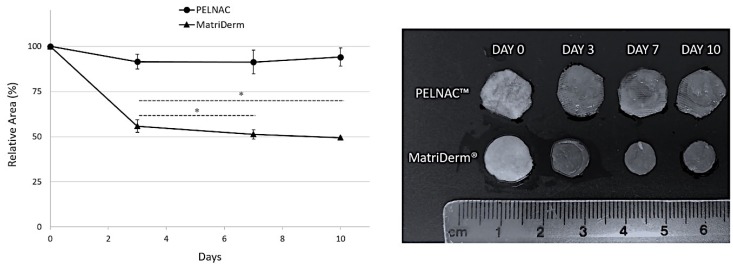
Matrix contraction data. While the size of PELNAC™ barely changed by 6% after ten days in culture, a significant reduction in the size of MatriDerm^®^ was observed. The bars on the graph denote the standard error of mean (*n* = 4). * *p* < 0.05.

Differences were observed between the two skin substitutes tested. While in the case of PELNAC™ the total area was barely reduced (~6%) after ten days of culture, for MatriDerm^®^ a significant reduction was observed with a relative area of ~51% after ten days in culture (Figure 6).

During experimentation, one of the materials (PELNAC™) adhered to the insert, making its removal with forceps difficult. In the case of MatriDerm^®^, upon removal of the weight, the biomaterial did not exert any resistance and was very easily removed with forceps. On many occasions, the biomaterial would lift off by itself. However, these were experimental observations and no measurements were taken to illustrate them.

### 3.4. Cell Proliferation

**Figure 7:**
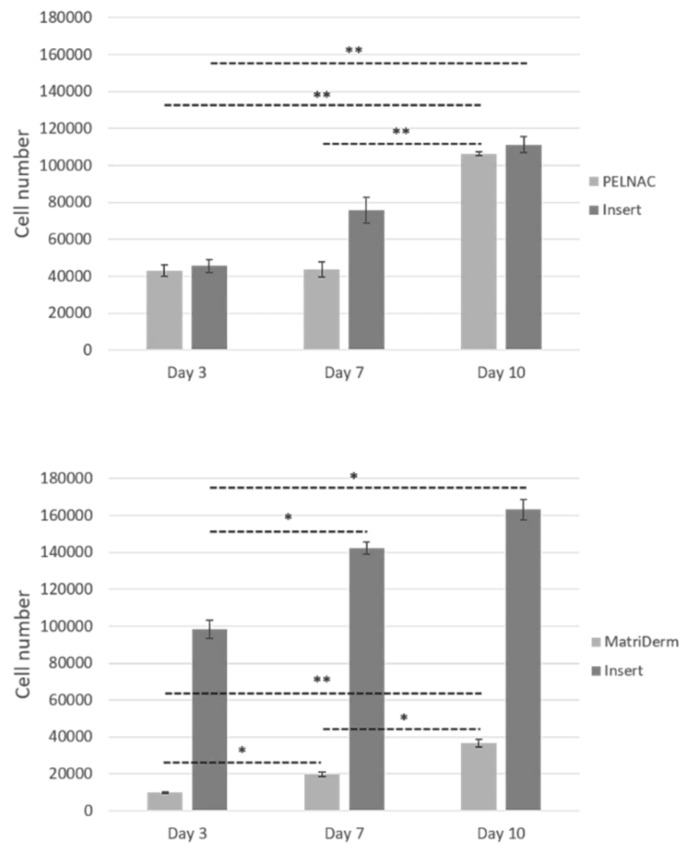
Cell proliferation by Alamar Blue assay. Results show average ± standard error of mean (*n* = 4). * *p* < 0.05. ** *p* < 0.001.

Cell proliferation was measured by Alamar Blue assay which showed statistically significant cell proliferation on the inserts as well as in the skin substitutes (Figure 7). Significant differences in cellular proliferation were observed between the two biomaterials tested. While there were more cells per biomaterial in the case of PELNAC™, there were more cells on the insert in the case of MatriDerm^®^: about 50% of the total cells were found in PELNAC™, whereas for MatriDerm^®^, the percentage of cells present in the biomaterial did not reach 20% of the total number of cells. After 10 days, the total number of cells (biomaterial + insert) hardly varied between the two cases, but the number of cells in PELNAC™ was three times greater than that of MatriDerm^®^. These results suggest that cells present in the inserts were able to infiltrate the biomaterials and that the degree of infiltration was dependent on the material used.

### 3.5. Cell Infiltration into Skin Substitutes

**Figure 8:**
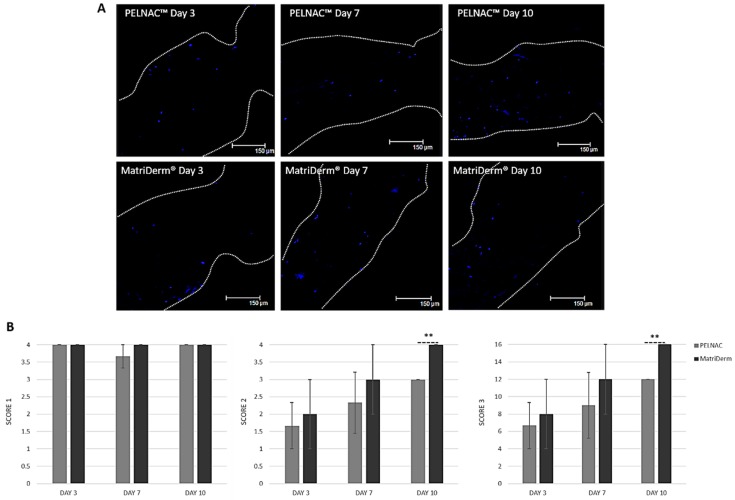
Cell infiltration into the scaffolds. (**A**) Representative confocal microscopy images of cells in scaffolds (white dotted line indicates edges of scaffold and blue fluoresce is indicative of cell nuclei). (**B**) Quantification of cell infiltration into the scaffolds: score 1 denotes distance migrated by the cells, score 2 denotes cell density and score 3 = score 1 × score 2. Results show average ± standard error of mean (*n* = 3). ** *p* < 0.001.

Results showed a progressive infiltration of cells into the scaffolds and confirmed that fewer cells were able to infiltrate MatriDerm^®^ compared to PELNAC™. The distance migrated by the cells (score 1) reached the whole width of the scaffolds from day 3. Interestingly, cells infiltrated the entire depth of MatriDerm^®^ more rapidly than in PELNAC™ and by day ten an even distribution of cells could be seen for MatriDerm^®^ (Figure 8).

### 3.6. Secretion of Exogenous Cytokines and Growth Factors

**Figure 9:**
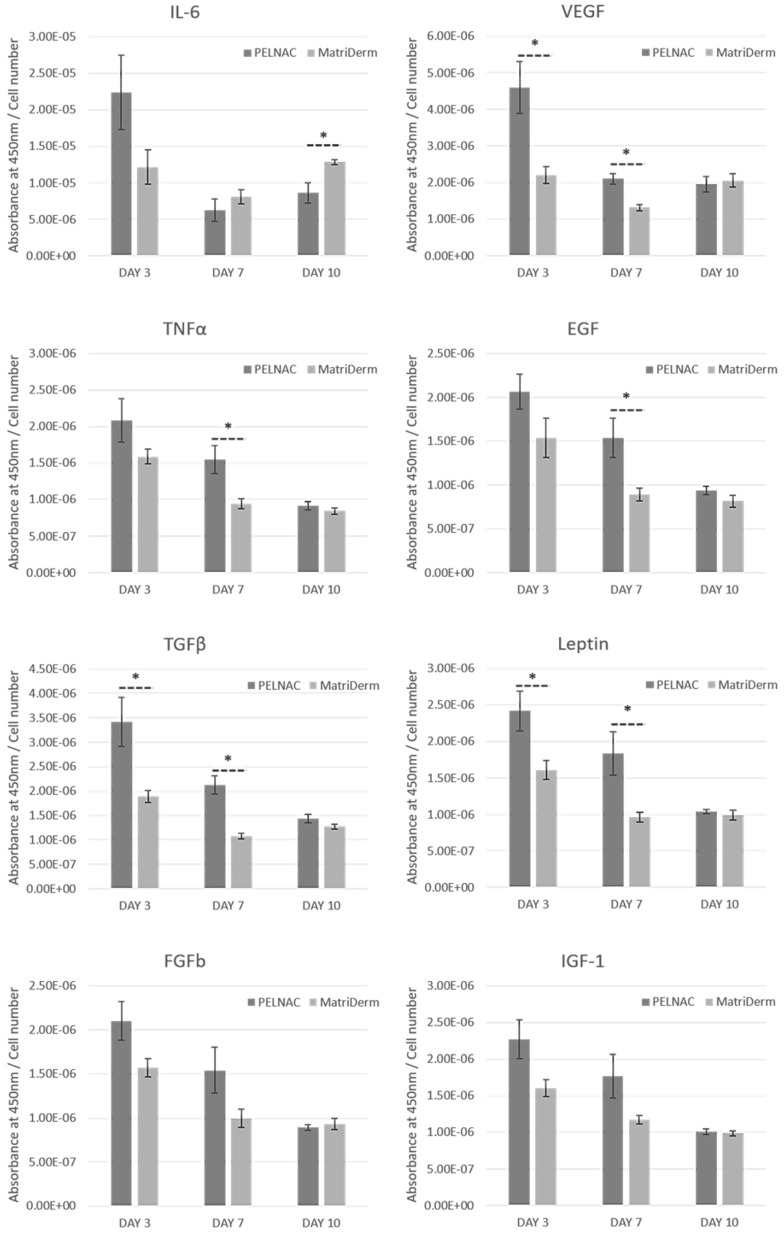
Secretion of exogenous cytokines and growth factors. Results show average ± standard error of mean (*n* = 4). * *p* < 0.05.

For the purpose of this study, we were interested in investigating whether differences in the secretion of exogenous cytokines and growth factors commonly involved in the process of wound healing could be observed between the two different groups (inserts with PELNAC™ and inserts with MatriDerm^®^) investigated with our model. Results were normalized per cell number and showed differences between the two groups (Figure 9). For most factors, secretion was maximum at day three and then it subsequently decreased by days seven and ten. Only secretion of IL-6 and VEGF followed a different trend for MatriDerm^®^ with minimum secretion on day seven and very similar secretion on days three and ten. Except for IL-6, secretion was higher for PELNAC™ on days three and seven, and similar for both groups on day ten. The exception was IL-6 as its secretion was higher for MatriDerm^®^ on days seven and ten. These results suggested that our proposed model can elicit and detect differences in the secretion of exogenous cytokines and growth factors between different scaffold treatments.

## 4. Discussion

Although many skin substitutes are clinically available, a complete functional skin substitute has yet to be produced [3]. Therefore, there is a clinical need to develop novel skin biomaterials; indeed, much effort is currently being directed towards such research. Part of the development process involves rigorous cell in vitro testing, which is currently done by simply pipetting a cell suspension on top of the 3D scaffold followed by static culture. However, this common experimental setup does not effectively reproduce the in vivo situation in which cells infiltrate the implanted biomaterial from the wound bed and edges. Our aim was to engineer a novel biomimetic in vitro model that mimics the natural cell infiltration process into scaffolds occurring during wound healing, therefore offering a realistic approach when testing novel skin biomaterials. For our work, we chose two commercially available and clinically well-established skin substitutes that, although both collagen-based (the vast majority of clinically used dermal materials are made of collagen since this is the main component of the dermal ECM), differ in their composition and structure. Furthermore, PELNAC incorporates a silicone backing, which many dermal materials do as a protective layer and for easy handling [1]. This way, we tested both a single-layered and a double-layered material representative of currently available dermal scaffolds [1]. MatriDerm^®^ is used as a dermal substitute in the treatment of, for example, full-thickness burns. It is used in a single stage operative procedure simultaneously with a split-thickness skin graft because of increased vascularization and elasticity. However, more scientific and clinical data is needed to verify its efficacy [3,12]. PELNAC™ is indicated for third degree burns, after surgical excisions of tumors, and at donor sites of skin flaps. It is used in a single or two-stage operative procedure, where rapid formation of neodermis has been reported before placement of the split-thickness skin graft [3,12].

The first step in engineering our model was to gelatin-coat the inserts, which was achieved using a straightforward protocol. A simple Coomassie blue staining of the implants confirmed that the protein coating had been deposited on the walls as well as on the bottom of the inserts. The gelatin coating mimics the natural collagen-rich ECM of the dermis. Furthermore, gelatin is a hydrolised version of collagen with exposed RGD binding sites which promote increased cellular attachment [8].

Secondly, it was important to achieve an even seeding of cells not only at the bottom of the inserts, but also on the cylindrical walls. For this purpose, we used a rotating seeding technique whereby cells were seeded at four locations (separated by 90° from each other) as well as at the bottom of the insert. Optimising time of incubation after completing seeding was important to ensure cell attachment without giving cells time to deposit an ECM and become embedded in it, which would have made cell migration into the scaffolds difficult. We found that periods of incubation longer than one hour significantly reduced cell infiltration into the biomaterials.

Finally, after placement of the scaffolds, it was necessary to ensure contact between the materials and the cell-seeded inserts, which we achieved by using a “weight” placed on top of the biomaterial.

Our engineered model allowed evaluation of several features relevant in wound healing, namely: matrix contraction, cellular infiltration into the biomaterial, cell proliferation inside the material, secretion of exogenous cytokines and growth factors, and interestingly, integration of the material with the surrounding tissue.

For successful implantation of skin substitutes, no scaffold contraction should occur [15]. Moreover, dimensional instability and changes in the porosity of scaffold can negatively affect cell attachment and infiltration [16]. Therefore, being able to predict the future behavior of skin substitutes in terms of contraction would be of utmost importance. Our study showed that matrix contraction can be measured using our model, which showed differences between the biomaterials tested.

Searching the scientific literature, we could only find a single study investigating PELNAC™ contraction without the silicone film [17]. In this work, the contraction ratios were much higher than those obtained in our study where PELNAC™ contained the silicone layer. They compared the effect of the presence or absence of fibroblasts on the contraction ratio of the biomaterial. The results showed that immersion of PELNAC™ in culture medium without fibroblasts already produced a contraction superior to that detected in our study. When the conditions of the experiment included fibroblasts grown on PELNAC™, the contraction was even higher than without cells [17]. Therefore, it may be speculated that the silicone layer in PELNAC™ stabilizes the matrix and prevents excessive contraction of the material.

In the case of MatriDerm^®^, in vitro or in vivo contraction studies were not found in the literature. When MatriDerm^®^ is applied in vivo, it is secured with sutures, staples or fibrin glue [18,19,20], thus preventing contraction of the material. The contraction results obtained in our study could be as a result of the absence of crosslinking and the lyophilization process of the material [1,9]. Interestingly, in an earlier study by our laboratory using a static culture system, contraction of MatriDerm^®^ when cultured with dermal fibroblasts was not observed, even though a much higher number of fibroblasts (5 × 10^5^) were seeded into the material [4]. This would suggest that our biomimetic model can better predict material contraction than static culture.

In terms of material structure, SEM images showed that the sponge of PELNAC™ is different to the matrix of MatriDerm^®^. Both materials are highly porous, 80–95% porosity for PELNAC™ and 98.20% porosity for MatriDerm^®^ according to the literature [11,21]. The two scaffolds have pores of different sizes (70–110 µm for PELNAC™ and 25–60 µm for MatriDerm^®^) that are interconnected. The contraction of MatriDerm^®^ may have negatively affected cell infiltration [16], not only because pore size would be affected, but also because contact between the insert walls and the biomaterial was lost.

In terms of the composition of the biomaterials, both are based on freeze-dried collagen. MatriDerm^®^ is composed of bovine collagen type I, III and V coated with 3% bovine elastin hydrolysate, whereas PELNAC™ is composed of porcine tendon-derived atelocollagen type I. Collagen is produced as procollagen, containing propeptides in the N and C-termini. Upon secretion into the ECM, the propeptides are cleaved, exposing the telopeptides that are adjacent to the collagenous domain and initiating collagen assembly into large highly organized supermolecular fibril structures [22,23]. Atelocollagen is a derivative of collagen obtained by removal of N- and C-terminal telopeptide components [13]. The cleavage of the collagen fibril telopeptides is critical for fibril turnover, tissue repair, and development since new and important interaction sites become available [22]. Therefore, the different biomaterials may present different binding sites that could also affect cell attachment and migration. Interestingly, cells populated the entire depth of MatriDerm^®^ sooner than in PELNAC™, which could be due to the reduced size of MatriDerm^®^ as a result of contraction.

Consequently, we believe that the higher cell proliferation observed in PELNAC™ compared to MatriDerm^®^ may be because of increased cell attachment, spreading, and infiltration, benefited by the absence of contraction, suggesting that when implanted in vivo the influx of cells into a material like PELNAC™ would be higher than into a material like MatriDerm^®^ [4].

As mentioned in the results, while PELNAC™ adhered to the insert, making its removal with forceps difficult, MatriDerm^®^ did not exert any resistance and was very easily removed with forceps. This difference may be a result of two different mechanisms. On the one hand, this adherence is affected by the different number of cells present in the biomaterials. A greater number of cells imply a greater secretion of ECM and therefore, a greater cohesion between the biomaterial and the insert. On the other hand, the integration is also affected by the different contraction rates of the scaffolds, because scaffold contraction disturbs the newly formed ECM and the separation between the biomaterial and the insert is favored.

Results showed differences in the secretion of exogenous cytokines and growth factors involved in wound healing [24,25] between the two scaffold treatments. Overall, PELNAC™ had higher secretions, particularly on days three and seven. IL-6 was the exception with MatriDerm^®^ obtaining higher secretions on days seven and ten. IL-6 is a pro-inflammatory cytokine that is important for timely wound healing. IL-6–deficient transgenic mice were shown to display significantly delayed cutaneous wound healing compared with control, wild-type animals [26]. This delayed wound healing was characterised by reduced inflammation, minimal epithelial bridge formation, and decreased granulation tissue formation [26].

Because the PELNAC™ supernatants had significantly higher VEGF titer at three and seven days incubation, compared to MatriDerm^®^, it may be predicted that the higher VEGF could lead to increased initial angiogenesis when this scaffold is used in vivo.

Expression of TGFβ family members is important in various aspects of wound healing such as inflammation, angiogenesis, reepithelialization, matrix formation and remodeling, and connective tissue regeneration [25]. It has been shown that TGFβ1 helps in the recruitment of additional inflammatory cells and that monocytes transform into macrophages in the wound by the influence of TGFβ among other factors [25,27]. Macrophages are important for clearing the wound of debris and microbes and release important cytokines and growth factors, that include TGF-β, which initiate the formation of granulation tissue [27]. TGFβ’s ability to stimulate collagen production is well documented. However, its over-expression is associated with pathological scarring [25]. Using our model, secretion of TGFβ was significantly higher for PELNAC™ at early time points, indicating that collagen production would likely be increased with a treatment of PELNAC™ compared to MatriDerm^®^.

Leptin is a mediator of wound reepithelialization [26]. Because PELNAC™ had significantly higher leptin content at three- and seven-days incubation, this scaffold type may produce better reepithelialization. The pro-inflammatory cytokine TNFα was significantly higher at day seven of culture for PELNAC™ than for MatriDerm^®^. Similar results were observed for EGF. Finally, although not significant, the same trend was observed for FGFb and IGF-1.

Overall, our results showed that our engineered model is able to induce and detect subtle differences in the secretion of factors involved in wound healing between different scaffold treatments. Static culture also allows differences in the secretion of these factors to be observed [4]. However, in static models secretion of factors is derived only from cells in the biomaterials. Therefore, it is not known how the biomaterial would affect secretion of factors from cells in the surrounding tissue. Our model allows the secretion of factors from cells in the biomaterial as well as in the insert (mimicking surrounding tissue), thus offering a more complete and realistic picture.

Based on the work presented in this paper, we believe that our model can be adapted to mimic the in vivo reality of wound healing even more accurately. Co-cultures could be incorporated, with keratinocytes, endothelial cells and immune cells (e.g., macrophages, leukocytes), as examples. One option could be to grow keratinocytes on the walls of the insert and HDF on the bottom, which would produce the growth factors required by the keratinocytes [28]. At the same time, endothelial cells could be grown at the bottom of the well plate. Patterns of leukocyte infiltration into the wound could be studied using our bio-constructs [29]. These would not maintain direct contact with the keratinocytes or HDF due to the membrane of the insert but could participate in cell signaling. Cell integration with the surrounding tissue could be quantified using mechanical pull-out testing. This model could be useful to study the effect of added therapeutic molecules and metabolites as growth factors or exosomes. Finally, the model could be miniaturised for high throughput screening and cost savings. We believe that our model could be used to not only test scaffolds under development but also to study molecular and cellular mechanisms that are important in the wound healing process.

## 5. Conclusions

We engineered a novel biomimetic in vitro model that mimics the natural cell infiltration process into scaffolds occurring during wound healing. This model offers a realistic approach when testing novel skin biomaterials under development. Using two different commercially available dermal substitutes, MatriDerm^®^ and PELNAC™, we showed that the model allows measurement and evaluation of several features relevant to the wound healing process, namely matrix contraction, cell infiltration and proliferation, integration of the biomaterial with the surrounding tissue, and secretion of exogenous cytokines and growth factors. Moreover, the model allows room for improvements or “adds-on”, such as co-culture, addition of therapeutic factors such as exosomes or growth factors, and miniaturization for scalability. This novel model can contribute to the development of functional skin substitutes for the treatment of burns and chronic wounds.
